# Optimizing delivery of an anti-cytomegalovirus inhibitory peptide using a cell-penetrating peptide

**DOI:** 10.1099/jgv.0.002210

**Published:** 2026-01-13

**Authors:** Komal Beeton, Jacob P. Haskell, Dipanwita Mitra, Erin B. Taylor, Gene L. Bidwell III

**Affiliations:** 1 Center for Immunology and Microbial Research, Department of Cell and Molecular Biology, University of Mississippi Medical Center, 2500 North State Street, Jackson, MS 39216, USA; 2 Department of Neurology, University of Mississippi Medical Center, 2500 North State Street, Jackson, MS 39216, USA; 3 Department of Physiology and Biophysics, University of Mississippi Medical Center, 2500 North State Street, Jackson, MS 39216, USA; 4 Department of Pharmacology and Toxicology, University of Mississippi Medical Center, 2500 North State Street, Jackson, MS 39216, USA

**Keywords:** antivirals, cell-penetrating peptide, cytomegalovirus, elastin-like polypeptide, pp150

## Abstract

Cytomegalovirus (CMV) is a beta herpesvirus that persists quiescently in healthy individuals but causes severe disease in immunocompromised patients and congenitally infected newborns. Side effects posed by the currently available antivirals necessitate the development of new antivirals with improved safety profiles. This study aims to optimize the potency of an anti-CMV peptide (P10) mimicking conserved region 2 of tegument pp150, fused with an elastin-like polypeptide (ELP). Our previous study has demonstrated that ELP-P10 inhibits murine cytomegalovirus (MCMV) growth *in vitro* and *in vivo* with enhanced pharmacokinetic properties relative to the free peptide. To enhance the potency of ELP-P10 at a lower concentration, this study utilizes a cell-penetrating peptide, SynB1, to facilitate the delivery of ELP-P10 into the cells. SynB1 was added to the N terminus of ELP-P10 to generate SynB1-ELP-P10. Antiviral efficacy and cytotoxic effects of ELP-P10 and SynB1-ELP-P10 were studied in cell culture. Pharmacokinetics, biodistribution and antiviral efficacy were studied in a mouse model of CMV infection. While ELP-P10 maintained significant antiviral activity against human cytomegalovirus (HCMV) in cell culture at a higher concentration, SynB1-ELP-P10 shows potency against HCMV and MCMV at a threefold lower concentration compared to ELP-P10. SynB1-ELP-P10 had similar bioavailability after subcutaneous administration as ELP-P10, and SynB1 conjugation to ELP-P10 significantly enhanced its accumulation in the kidneys. Moreover, in an *in vivo* model of CMV infection, ELP-P10 and SynB1-ELP-P10 treatment led to a significant reduction in the viral titre compared to controls. In conclusion, the strategic modification of ELP-P10 with SynB1 potentiated CMV inhibition, allowing for the use of lower therapeutic doses and mitigating potential side effects.

## Introduction

Human cytomegalovirus (HCMV) infects most people in the world, causing benign or self-limiting infection in healthy individuals [1,3]. Both primary infection and reactivation of the latent virus are often clinically silent in immunocompetent individuals [4,7]. However, it is a cause of concern for organ transplant recipients and immunocompromised patients, who can develop severe diseases such as retinitis, pneumonia and gastrointestinal disease [6,12]. Furthermore, congenital cytomegalovirus (cCMV) is the most common prenatal viral infection, affecting 0.5–2% of all live births [13,18]. An estimated 5–10% of congenitally infected newborns have hepatosplenomegaly, jaundice, microcephaly, intellectual disability or non-genetic sensorineural hearing loss [15,17,19].

Prophylactic antiviral drugs or pre-emptive therapies have been developed to treat HCMV infection. Currently available antivirals include ganciclovir (GCV), valganciclovir, foscarnet (FOS), cidofovir (CDV), acyclovir (ACV), valacyclovir, letermovir and fomivirsen, but their long-term use poses side effect/toxicity issues that limit their clinical application [25,27]. For instance, the use of GCV has been shown to induce thrombocytopenia, ACV can cause kidney damage and haemolytic uraemic syndrome and FOS and CDV possess nephrotoxicity issues [27,30]. During pregnancy, treating primary maternal HCMV infections with valacyclovir has been shown to effectively prevent foetal HCMV infection but is associated with a small risk of reversible maternal renal dysfunction [31,33]. Moreover, currently available treatments during cCMV infection, including GCV/valganciclovir or valacyclovir, show moderate improvement in audiological and neurodevelopmental outcomes but are associated with neutropenia and other cytotoxic effects and raise safety concerns for the mother and foetus [34,36]. Therefore, the lack of a vaccine and the side effects posed by currently available antivirals necessitate the development of novel therapeutics with improved safety profiles. One emerging class is the use of peptide therapeutics as antiviral drugs; for instance, the use of enfuvirtide, a peptide mimicking gp41 of human immunodeficiency virus (HIV), used in combination therapy for the treatment of HIV-1 [37,38]. Although peptide therapeutics possess pharmacokinetic challenges, conjugation of peptides with drug delivery molecules/carrier proteins or cell-penetrating peptides (CPPs) can help overcome these challenges.

In a previous study from our lab, a 9 aa peptide (P10) targeting HCMV tegument protein pp150 (basic phosphoprotein 150) was designed to inhibit virus maturation [39]. pp150 is a tegument protein that controls cytoplasmic events during cytomegalovirus (CMV) maturation by stabilizing the nucleocapsid organization during secondary envelopment in the virus assembly compartment [40,42]. By engaging the conserved regions 1 and 2 (CR1 and CR2) on its N terminus, pp150 interacts with the capsid proteins to stabilize the nucleocapsids to produce infectious virions [43]. P10 is a 9 aa peptide mimicking the CR2 of pp150 that was designed to competitively inhibit pp150-capsid interaction and thereby inhibit the virus maturation [39]. Treatment of HCMV- and MCMV-infected cells with P10 led to a reduction in HCMV and MCMV growth and spread in cell culture, paving the way for its development into an antiviral against CMV [39]. To overcome the challenges associated with the *in vivo* use of peptide therapeutics, P10 was conjugated with a drug delivery system using the elastin-like polypeptide (ELP) platform. ELP is a protein derived from a repeated sequence found in human tropoelastin [44]. ELPs are optimal as drug carriers because they stabilize small-molecule or peptide cargo in circulation [45]. They are inert and thus are likely inherently safe, biodegradable and non-immunogenic. Most importantly, ELPs do not cross the placental barrier, making them an ideal drug delivery system for use during pregnancy to sequester drugs in maternal circulation and prevent exposure to the developing foetus [46,47]. Therefore, we chose ELP for the delivery of the P10 peptide. Our previous study using ELP-conjugated P10 (ELP-P10) showed that ELP-P10 maintained significant antiviral activity in cell culture against murine cytomegalovirus (MCMV) [48]. Pharmacokinetic and biodistribution studies using a mouse model showed that conjugation of P10 with ELP enhanced its plasma half-life with a terminal half-life of ~6.31 h compared to ~1.9 h for the free peptide following a subcutaneous injection [48]. ELP-P10 showed higher accumulation in the mouse liver and kidneys compared to unconjugated P10. Moreover, in a mouse model of MCMV infection, ELP-P10 treatment led to a significant reduction in the viral titre compared to the unconjugated P10 [48].

Our previously published data show that ELP-P10 is a promising candidate for the development of an effective antiviral therapy against CMV infection; one potential limitation is its half-maximal inhibitory concentration (IC_50_) against MCMV, which was ~20-fold higher compared to unconjugated P10. To address this limitation, we used the CPP SynB1 to enhance the delivery of ELP-P10 into the cells. CPPs are 5–30 aa peptides that are known to mediate the uptake of the attached proteins/peptides by endocytosis, endosomal uptake or by direct penetration into the plasma membrane [49,52]. SynB1 is a CPP derived from protegrin and is known to mediate the cellular uptake of its cargo via endocytic mechanisms [53,54]. In this study, SynB1 was attached to the N terminus of ELP-P10 to generate the SynB1-ELP-P10 construct. We hypothesize that SynB1 modification will enhance the delivery of ELP-P10 into the cell, thereby improving the potency and enhancing the overall antiviral efficacy compared to the unmodified ELP-P10. The SynB1-ELP-P10 fusion protein was designed, expressed and purified to compare its *in vitro* cellular uptake and localization to the parent ELP-P10 construct, and the inhibitory activity and the IC_50_ of SynB1-ELP-P10 were quantified against HCMV and MCMV *in vitro*. Furthermore, the impact of SynB1 modification on the pharmacokinetics and biodistribution of ELP-P10 was assessed in a mouse model, and its efficacy to reduce viral load was tested in a mouse model of CMV infection.

## Methods

### Generation of SynB1-ELP-P10 construct

**Fig. 1.**
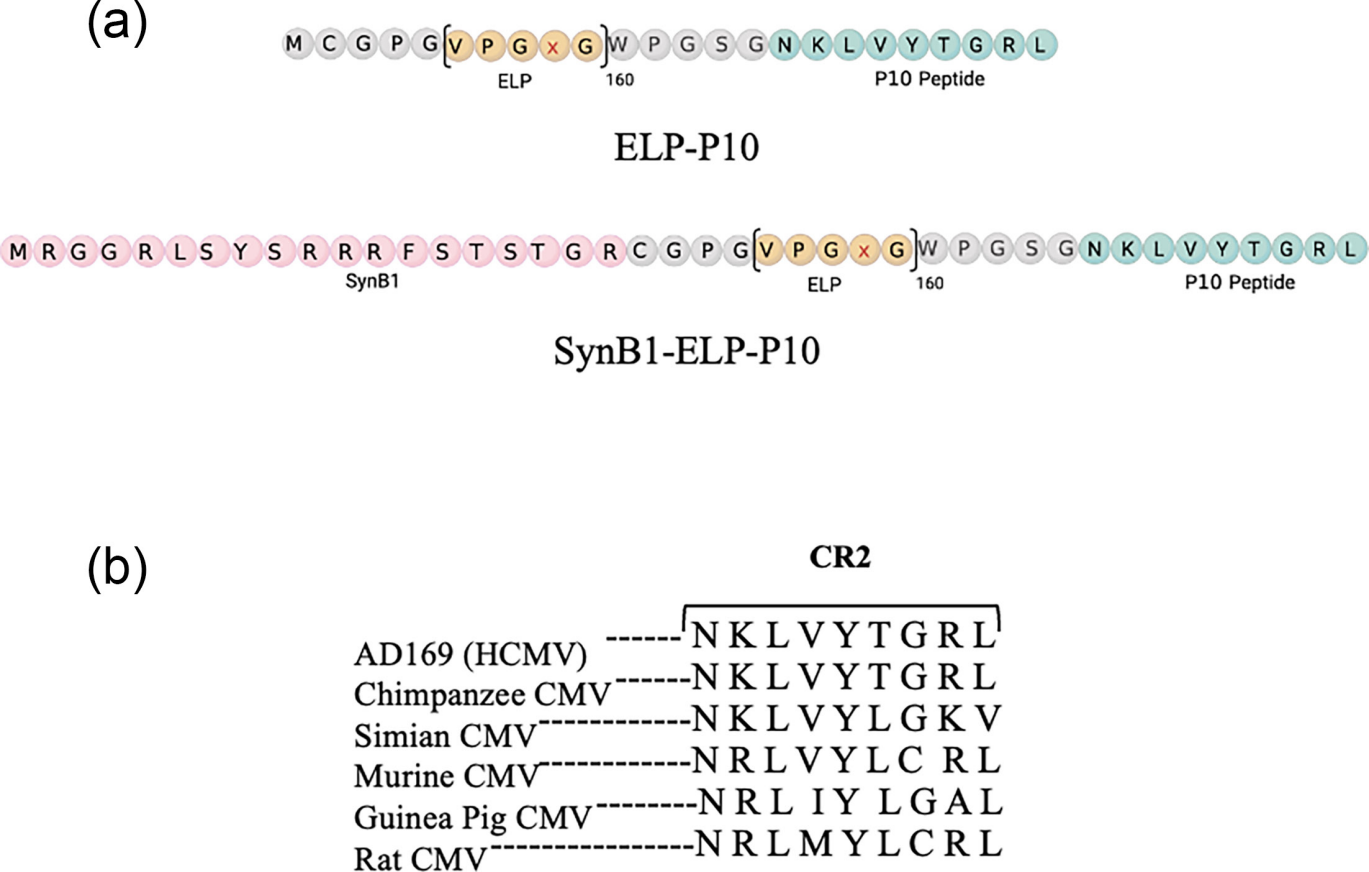
(**a**) Illustration of the peptide constructs: ELP-P10 peptide (top) and SynB1-conjugated ELP-P10 (bottom). A CPP, SynB1, was added to the N terminus of ELP-P10 to generate SynB1-ELP-P10. (**b**) Conservation of the CR2 in beta herpesviruses: sequence alignment using the predicted protein sequences of HCMV, chimpanzee CMV, simian CMV, MCMV, guinea pig CMV and rat CMV using clustalW shows the conservation of the CR2 in beta herpesviruses.

To make the SynB1-ELP-P10 construct (Fig. 1a), pET25b vectors containing SynB1-Sfi-P9, a construct that contains the SynB1 CPP and an unrelated Zika virus inhibitory peptide called P9 separated by an SfiI restriction site, and cys-Sfi-P10, a construct containing the CMV inhibitory P10 peptide but lacking the SynB1 peptide, were digested with enzymes PstI and SfiI, the appropriate DNA fragment from each vector was gel purified and the SynB1- and P10-containing fragments were ligated to yield a SynB1-Sfi-P10 construct. Next, an SfiI digest was conducted for SynB1-Sfi-P10 to ligate the ELP sequence, removed from a separate plasmid by compatible restriction enzymes, at the SfiI site to produce SynB1-ELP-P10. The ligated product was transformed into XL1 blue competent cells and plated on agar plates containing ampicillin. Bacterial colonies containing the construct were confirmed by DNA sequencing. This DNA was further transformed to Clear Coli^®^ for protein expression, and proteins were purified as explained previously [48]. A control protein lacking the P10 peptide (SynB1-ELP) was also purified in a similar manner.

### Cells and viruses

The cells used in this study were low-passage human foreskin fibroblasts (HFFs, passage 6 to 13) and mouse embryonic fibroblasts (MEFs). HFFs and MEFs were cultured in complete Dulbecco’s modification of Eagle’s medium (DMEM with 4.5 g l^−1^ glucose, l-glutamine and 1 mM sodium pyruvate) supplemented with 10% heat-inactivated FBS, 2 mM l-glutamine and 100 U ml^−1^ penicillin/streptomycin. All cells were grown at 37 °C and 5% CO_2_. HCMV Towne BAC was used to infect HFFs, and MCMV K181 was used for MEF infections. Towne BAC and K181 were cultured on HFFs and MEFs, respectively, and titered using plaque assay.

### Viral infections

For infections, the media was removed from the cell culture plates, followed by incubation with the appropriately diluted virus stock in the raw DMEM for 1 h. After 1 h, the media containing the virus was removed, and the cells were washed three times with PBS and incubated with the complete DMEM until the endpoint.

### Half-maximal inhibitory concentration

IC_50_ of ELP-P10 and SynB1-ELP-P10 was determined using the plaque reduction assay. For determining the IC_50_ against HCMV, HFFs were grown in six-well plates in triplicates. Upon confluency, the cells were infected with Towne BAC at an m.o.i. of 0.01 for 1 h. After 1 h, the infection media was removed, and cells were washed three times with PBS and then incubated with twofold serially diluted ELP-P10 (concentration range 200–1.5 µM) or ELP (control) in complete DMEM for 5 days. At 5 days post-infection (dpi), the supernatant and the cells were harvested and stored at −80 °C to be used for plaque assay. For the IC_50_ of SynB1-ELP-P10, a concentration range of 25–1.5 µM was used.

For the IC_50_ of SynB1-ELP-P10 against MCMV, MEFs were grown in six-well plates in triplicates and infected with MCMV K181 at an m.o.i. of 0.01 for 1 h. After 1 h, the infection media was removed, and cells were washed three times with PBS and then incubated with twofold serially diluted SynB1-ELP-P10 (25–1.5 µM) in complete DMEM for 3 days. After 3 dpi, infected cells and supernatant were harvested and stored at −80 °C to be used for plaque assay. All the IC_50_ experiments were performed in triplicate and repeated as three independent experiments.

### Viral titre using the plaque assay

To determine the viral titres in HCMV- and MCMV-infected cells/mice organs, monolayers of HFFs/MEFs were grown in 12-well plates in triplicate and infected with serial dilutions of the infected samples (10^−1^–10^−3^) in DMEM for 1 h. At 1 h post-infection, the infection media was removed, and cells were washed with PBS twice and incubated with complete DMEM containing gamma immunoglobulin for 10 days for HCMV-infected cells. MCMV-infected cells were overlayed with pre-warmed CMC and complete DMEM in a 1:4 ratio and incubated for 3 days. At the endpoint (10 days for HCMV, 4 days for MCMV), the media was removed, and cells were washed twice with PBS, followed by methanol (100%) fixation for 7 min. For staining, the HCMV-infected cells were stained with Giemsa stain (Sigma-Aldrich, Milli-pore Sigma, Burlington, MA, USA, catalogue no. GS1L) for 15 min. The MCMV-infected cells were stained using 1% crystal violet for 30 min. The plates were then washed with tap water, air-dried, and HCMV and MCMV plaques were quantified.

### Plaque size comparison

For plaque size comparison, cells were infected with HCMV at a low m.o.i. of 0.01 and followed by continuous treatment with 80 µM (IC_75_ of ELP-P10 used previously [48]) ELP-P10, ELP control and P10 for 1 h before infecting them with HCMV at a low m.o.i. of 0.01. Cells were fixed at 10 dpi, and virus yield was measured by counting the number of plaques. Individual plaque sizes were measured by counting the area of the plaques in ImageJ software (National Institutes of Health, Bethesda, MD, USA). Data were analysed by Student’s t-tests comparing the means of the test and the control group. The sem was plotted as error bars. A *P* value of <0.05 was considered significant.

### Cell viability

Cell viability was measured using CellTiter 96^®^AQ_ueous_ One Solution Cell Proliferation Assay (Promega). HFFs and MEFs were grown in 96-well plates in triplicate and infected with HCMV/MCMV or mock-infected at an m.o.i. of 1 for 1 h. At 1 h post-infection, the cells were incubated with 23.41 µM (a concentration equal to the IC_50_ of ELP-P10) of SynB1-ELP-P10/ELP-P10 or controls SynB1-ELP-/ELP-P10 dissolved in complete DMEM for one replication cycle (3 days for MCMV and 5 days for HCMV). At the endpoint, the cells were washed twice with PBS, followed by the addition of 20 µl of CellTiter 96^®^AQ_ueous_ One Solution Reagent in the 96-well plate containing complete DMEM and incubated for 2 h at 37 °C and 5% CO_2_. Absorbance was measured at 490 nm using Tecan’s Magellan Microplate Reader.

### Rhodamine labelling of ELP-P10 and SynB1-ELP-P10

ELP-P10 and SynB1-ELP-P10 were fluorescently labelled on a unique cysteine residue using tetramethylrhodamine-5-maleimide. Both proteins were diluted to 100 µM in Na_2_H_2_PO_4_ buffer. The diluted protein was then mixed with 1 mM TCEP [tris(2-carboxyethyl)phosphine] for 1 h while rotating at room temperature, followed by the addition of rhodamine to a final concentration of 200 µM. This mix was incubated overnight at 4 °C with continuous stirring to ensure the labelling of the proteins with rhodamine. Any remaining free dye was removed by thermal cycling [55]. Protein concentration and efficiency of labelling on the single cysteine residue were assessed by UV–Visible spectrophotometry by measuring absorbance at 280 and 541 as previously described [48].

### Confocal microscopy

For confocal microscopy, HFFs were grown on coverslip inserts in six-well tissue culture plates to confluency. Cells were incubated with rhodamine-labelled SynB1-ELP-P10 or ELP-P10 (10 µM) for 1 h in triplicates. At the endpoint (1, 4 and 24 h), media was removed, and cells were washed twice with PBS. Cells were fixed using 4% paraformaldehyde for 10 min, followed by staining with DAPI for 5 min. The fixed and stained cells were then mounted on the glass slides with a drop of PBS and air-dried before acquiring images with a laser scanning confocal microscope (Nikon) with a 60× oil immersion objective using 405 and 561 nm lasers to excite DAPI and rhodamine, respectively. Images were collected using NIS-Elements imaging software.

### Mice

Male and female BALB/c mice (6–9 weeks of age), maintained under pathogen-free conditions, were used for this study.

### Pharmacokinetics and biodistribution of ELP-P10 and SynB1-ELP-P10

BALB/c mice (female, *n*=4 per group) were anesthetized using isoflurane and were injected with rhodamine-labelled ELP-P10 or SynB1-ELP-P10 at a concentration of 500 nmol kg^−1^ subcutaneously. Blood was collected via the tail vein at regular intervals up to 72 h post-injection to calculate plasma concentration over time. Plasma was collected from centrifuged blood samples and analysed in the BioTek Cytation 7. The fluorescence of plasma samples was directly measured using a Take 3 Microvolume Plate (Agilent; Excitation: 535, Emission: 580, Gain: 100) and fit to a standard curve of known concentration of ELP-P10 and SynB1-ELP-P10. To determine pharmacokinetic parameters, plasma concentrations during the clearance phase (from the peak of the plasma concentration curve) were fit using a two-compartment pharmacokinetic model as described previously using GraphPad Prism [56]. For biodistribution analysis, mice (female, *n*=4 per group) were euthanized 4 h after injection with ELP-P10 or SynB1-ELP-P10 or saline control, and the brain, heart, liver, lungs, kidneys and spleens were harvested and imaged using the *In Vivo* Imaging System (IVIS, Perkin Elmer). Regions of interest were drawn around each organ using Living Image software (Perkin Elmer) to determine average radiant efficiencies for each organ. Autofluorescence values were determined from imaging organs from a saline-injected animal and were subtracted from the average radiant efficiency of each organ. The data were fit to a standard curve of known concentrations of SynB1-ELP-P10 or ELP-P10.

### *In vivo* efficacy in mice

Mice (3 males, 3 females per treatment group) were infected intraperitoneally with 1×10^6^ p.f.u. of MCMV K181. At 1 h post-infection, mice were anesthetized using isoflurane and subcutaneously injected with 1,000 nmol Kg^−1^ of the ELP-P10, SynB1-ELP-P10, SynB1-ELP or vehicle-treated. Animals were treated with the same dose of proteins every 24 h, and their weight was monitored. In brief, 3 dpi, animals were euthanized, and the liver, kidneys, spleen and heart were harvested and stored at − 80 °C. Virus titres in the harvested organs were determined by plaque assay.

### Peripheral immune cell analysis

Blood was collected from the animals at the conclusion of the study. The blood was centrifuged at 350 ***g*** for 5 min to isolate plasma. Erythrocytes were lysed by adding 10X volume of 1X PharmLyse (BD Biosciences, San Jose, CA). After incubation for 5 min at room temperature, the blood was centrifuged at 200 ***g*** for 5 min. The pelleted peripheral blood leucocytes (PBLs) were washed two times with 1X PBS and 2% FCS and centrifuged at 350 ***g*** for 5 min, and then immediately used in flow cytometric analyses. Cells were stained with 575V Viability stain (BD Biosciences) and then washed 2X with 1X PBS, 2% FCS and 0.9% sodium azide to remove unbound stain. Briefly, 1×10^6^ cells (100 µl) were aliquoted into a flow cytometry tube and incubated with 0.25 µg of anti-mouse CD32/CD16 (FcR block, BD Biosciences) for 5 min on ice. Cells were then stained with either isotype control antibodies or antibodies, CD45 BUV395 (clone 30-F11), CD45R APC (clone RA3-6B2), CD11b PE-Cy7 (clone M1/70), Ly6G-BV421 (clone 1A8), Ly6C BUV737 (clone HK1.4), CD3 BV650 (145-2C11), CD8 PerCP-Cy5.5 (clone 53-6.7), CD4 FITC (clone GK1.5), NK1.1 PE (clone PK136) for 30 min on ice protected from light. All antibodies were diluted 1:200 in 1X PBS containing 2% FCS and 0.09% sodium azide. All antibodies utilized for staining in these experiments have been extensively validated by the company (BD Biosciences). When possible, a positive and negative control staining sample is included to further confirm specificity. Samples were analysed on a BD FACSymphony A3 flow cytometer, and a total of 50,000 CD45+ cells were acquired for each sample. Data were analysed using FlowJo version 10.8.2.

## Results

### Generation of ELP-P10 chimeric fusion protein

To overcome the limitations of a higher IC_50_, we conjugated ELP-P10 with a CPP, SynB1. The SynB1-ELP-P10 chimeric fusion protein was constructed by appending the SynB1 peptide to the amino (N) terminus of the ELP-P10 (Fig. 1a). The ELP used in our study has a size of ~ 62 kDa with 160 repeats and a phase transition temperature of 65 °C, ensuring that the protein will not undergo phase transition and aggregation at physiological temperatures. P10 is an anti-CMV inhibitory peptide targeting the CR2 of pp150, which is highly conserved among beta herpesviruses (Fig. 1b).

### ELP-P10 treatment inhibits HCMV growth and spread in cell culture

**Fig. 2.**
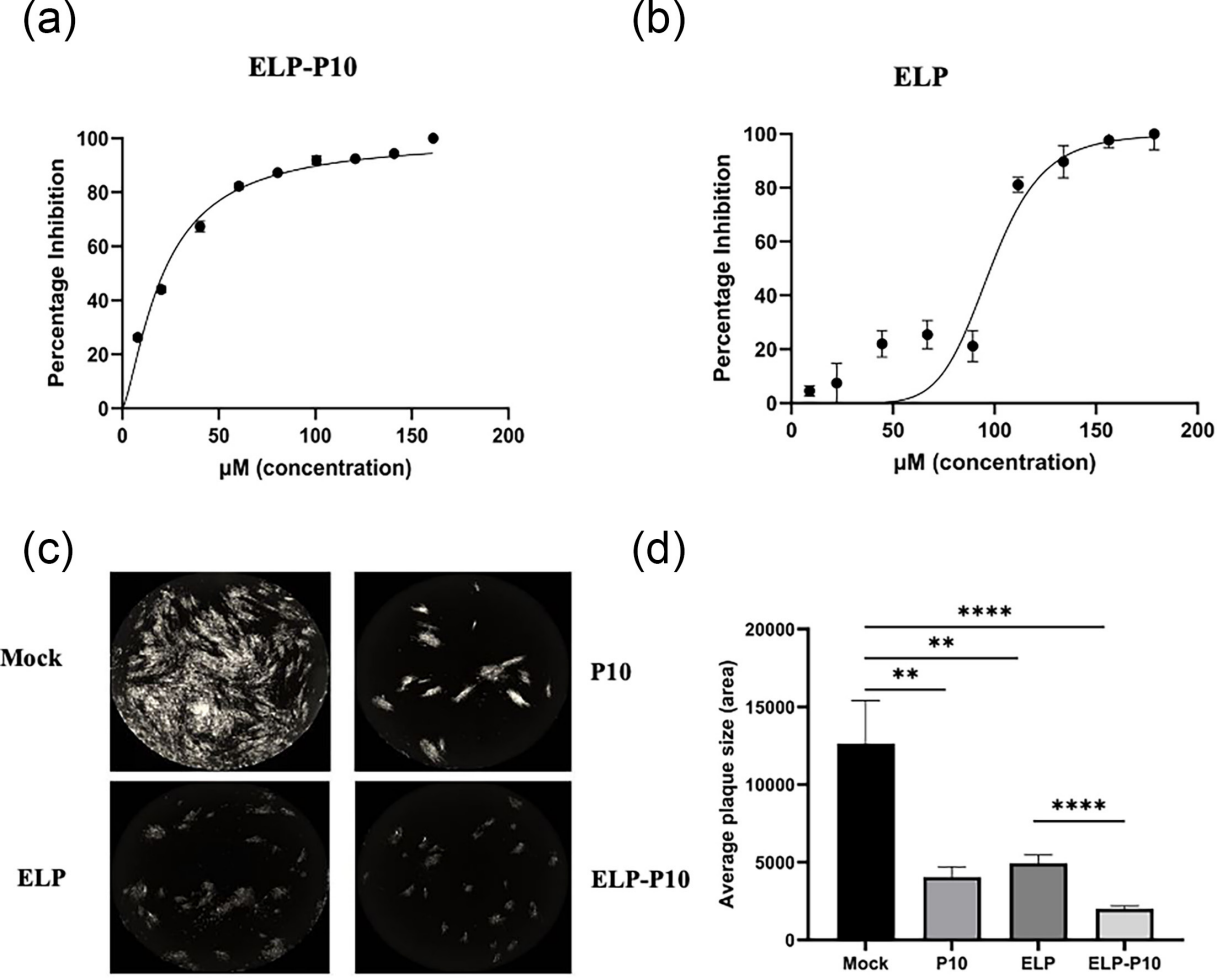
Assessing the inhibitory potential of ELP-P10 against HCMV in HFFs: the IC_50_ of (a) ELP-P10 and (b) ELP against HCMV was assessed by infecting cells at a low m.o.i. of 0.01 for 1 h, followed by treatment with ELP-P10 or ELP (control) for 5 days. The % inhibition of virus growth from three independent experiments performed in triplicate was plotted against the concentration. The IC_50_ of ELP-P10 was calculated to be at 21.33 µM with a 95% Cl [18.93, 23.63], whereas 98.04 µM with a 95% CI [87.69, 107.4] for ELP. (**c**) Images showing plaque size comparison among ELP-P10, ELP and P10-treated cells. Individual plaque sizes of 10 dpi were measured by counting the area of the plaques in ImageJ software. (**d**) Plaque size comparison in ELP-P10, ELP, P10-treated and no-treatment control groups. Data were analysed using Student’s t-tests to compare the means of the test and control groups from three independent experiments performed in triplicate. sem was plotted as error bars, and a *P* value of <0.05 was considered significant. ELP-P10-treated infected cells showed significantly smaller plaque sizes compared to the controls.

As P10 targets the CR2 of pp150, which is a CR in all beta herpesviruses, its antiviral potential can be assessed in both HCMV and MCMV models [42]. Our previous study established that ELP-P10 successfully inhibits MCMV growth at an IC_50_ of 23.41 µM [48]. In order to use ELP-P10 in clinical settings, it’s crucial to determine its efficacy against HCMV. Therefore, to determine if ELP-P10 is effective against HCMV, the antiviral efficacy of ELP-P10 against HCMV was tested. The IC_50_ of ELP-P10 against HCMV was determined by treating the HCMV-infected HFFs with varying concentrations of ELP-P10. The IC_50_ of ELP-P10 was calculated to be 21.33 µM with a 95% confidence interval (CI) [18.93, 23.63] (Fig. 2a). However, comparing the IC_50_ of P10 and ELP-P10, the data suggest that the ELP-delivered peptide is ~16X less potent than the free peptide [39].

Interestingly, as observed previously with MCMV, the ELP carrier alone inhibits HCMV, though only weakly. To show that the inhibition by ELP control is different from specific inhibition by ELP-P10, the IC_50_ of ELP control was also calculated. The IC_50_ of ELP control was estimated to be at 98.04 µM with a 95% CI [87.69, 107.4] (Fig. 2b). The results indicate that the IC_50_ of ELP control is significantly higher than that of ELP-P10 (21.23 µM), and the inhibitory effect of the ELP-P10 treatment group is considerably greater compared to that of the ELP control group. To further assess the impact of ELP control treatment on virus spread, plaque sizes were calculated and compared between ELP control versus ELP-P10-treated groups. ELP-P10-treated infected cells showed significantly smaller plaque sizes compared to the ELP control group, indicating a reduction in virus spread to the adjacent cells, hence inhibiting the virus growth (Fig. 2c, d).

### Addition of a CPP SynB1 enhances the potency of ELP-P10 against HCMV

**Fig. 3.**
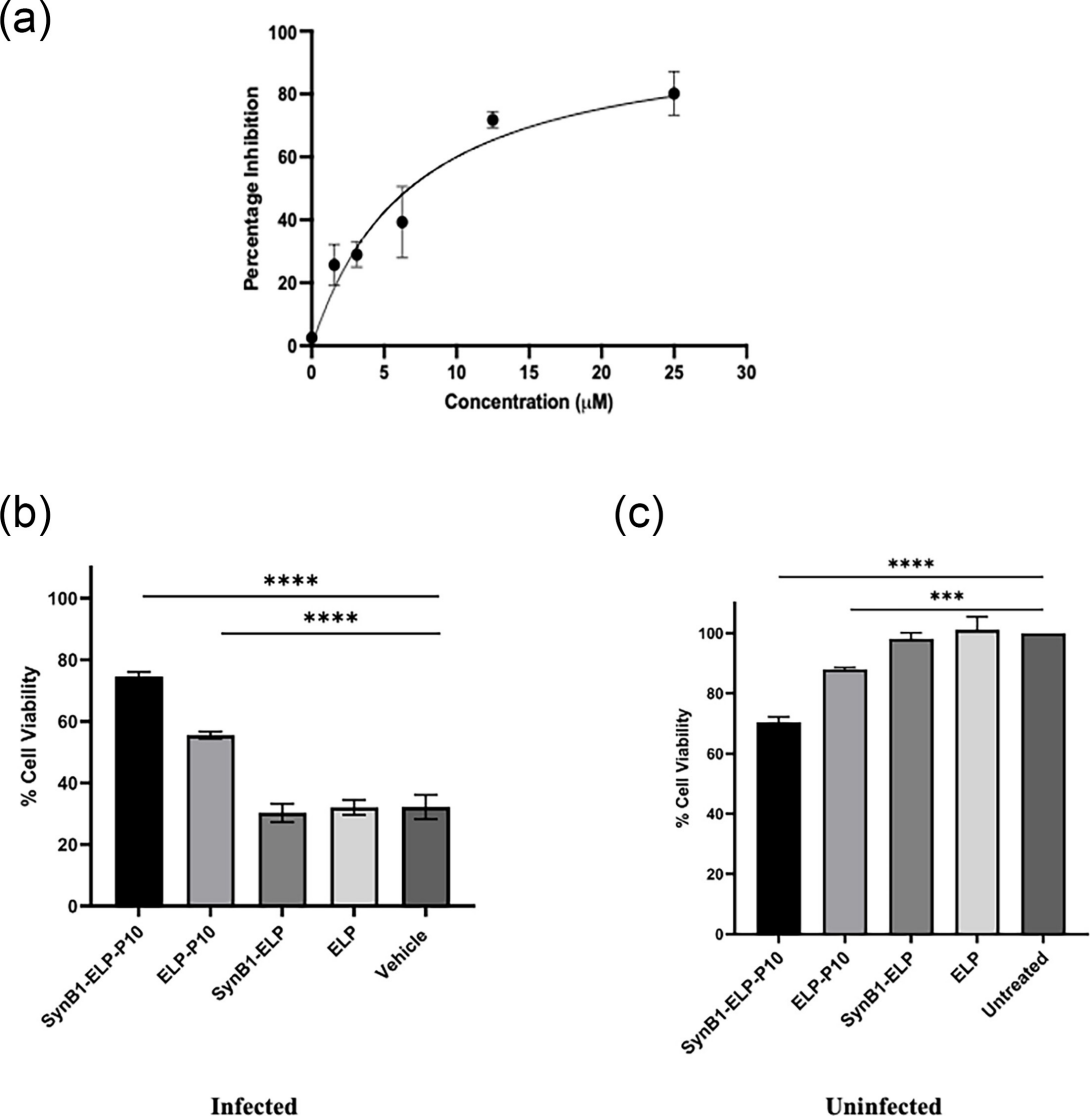
(**a**) Assessing the inhibitory potential of SynB1-ELP-P10 against HCMV in HFFs: the IC_50_ of SynB1-ELP-P10 against HCMV was assessed by infecting cells at a low m.o.i. of 0.01, followed by treatment with SynB1-ELP-P10 for 5 days. The IC_50_ of SynB1-ELP-P10 from three independent experiments performed in triplicate was calculated to be at 6.73 µM with a 95% CI [4.759–8.709]. Cell viability (%) in (b) HFFs infected with HCMV and (c) uninfected HFFs, along with different treatment groups. HFFs were infected with HCMV with an m.o.i. of 1 for an hour, followed by treatment with 23.14 µM of SynB1-ELP-P10 or ELP-P10 along with appropriate controls (SynB1-ELP-GGC or ELP) or vehicle-treated in triplicate for 5 days. Cell viability was assessed by MTS assay. The percentage of cell survival was plotted for different treatment groups, and the data were analysed by one-way ANOVA using the Tukey post-hoc test from three independent experiments performed in triplicates.

ELP-P10 treatment significantly reduced HCMV and MCMV growth in cell culture, but the IC_50_ was higher compared to unconjugated P10. To address this limitation, a CPP, SynB1, was incorporated with ELP-P10 to generate the SynB1-ELP-P10 construct. To test our hypothesis that SynB1 conjugation of ELP-P10 might reduce its IC_50_, the antiviral efficacy of SynB1-ELP-P10 was tested against HCMV. SynB1-ELP-P10 treatment led to a significant reduction in the viral titre in the infected cells with an IC_50_ of 6.73 µM with a 95% CI [4.759, 8.709] (Fig. 3a) compared to the SynB1-ELP (control) treatment, which did not show any effect on the viral loads. This shows that conjugation of ELP-P10 with SynB1 reduced the IC_50_ of ELP-P10 by more than threefold.

To rule out the possibility that the reduction in the viral titres could be due to the cytotoxic effects posed by SynB1-ELP-P10, cell viability of the infected and uninfected cells was determined using CellTiter 96^®^AQ_ueous_ One Solution Cell Proliferation Assay. As shown in Fig. 3(b), following HCMV infection, vehicle-treated infected cells had only 30% cell viability compared to uninfected cells, which indicates that HCMV infection is associated with lytic cell death. SynB1-ELP-P10 and ELP-P10 treatments led to higher cell viability compared to the vehicle. Moreover, SynB1-ELP-P10-treated cells demonstrated a significantly higher cell viability (75% viable cells) than ELP-P10 (50% viable cells) at equimolar concentration. SynB1-ELP and ELP control treatments had little to no effect on the cell viability of the infected cells compared to the vehicle. Although Syn-ELP-P10 and ELP-P10 treatment reduced the metabolic activity in the uninfected cells (Fig. 3c), it still showed a significant protective effect from HCMV infection. This demonstrates the specific inhibition of HCMV by SynB1-ELP-P10 and ELP-P10, with SynB1-ELP-P10 being more potent than ELP-P10.

### SynB1-ELP-P10 successfully inhibits MCMV growth in cell culture and prevents the infected cells from virus-induced lytic cell death

**Fig. 4.**
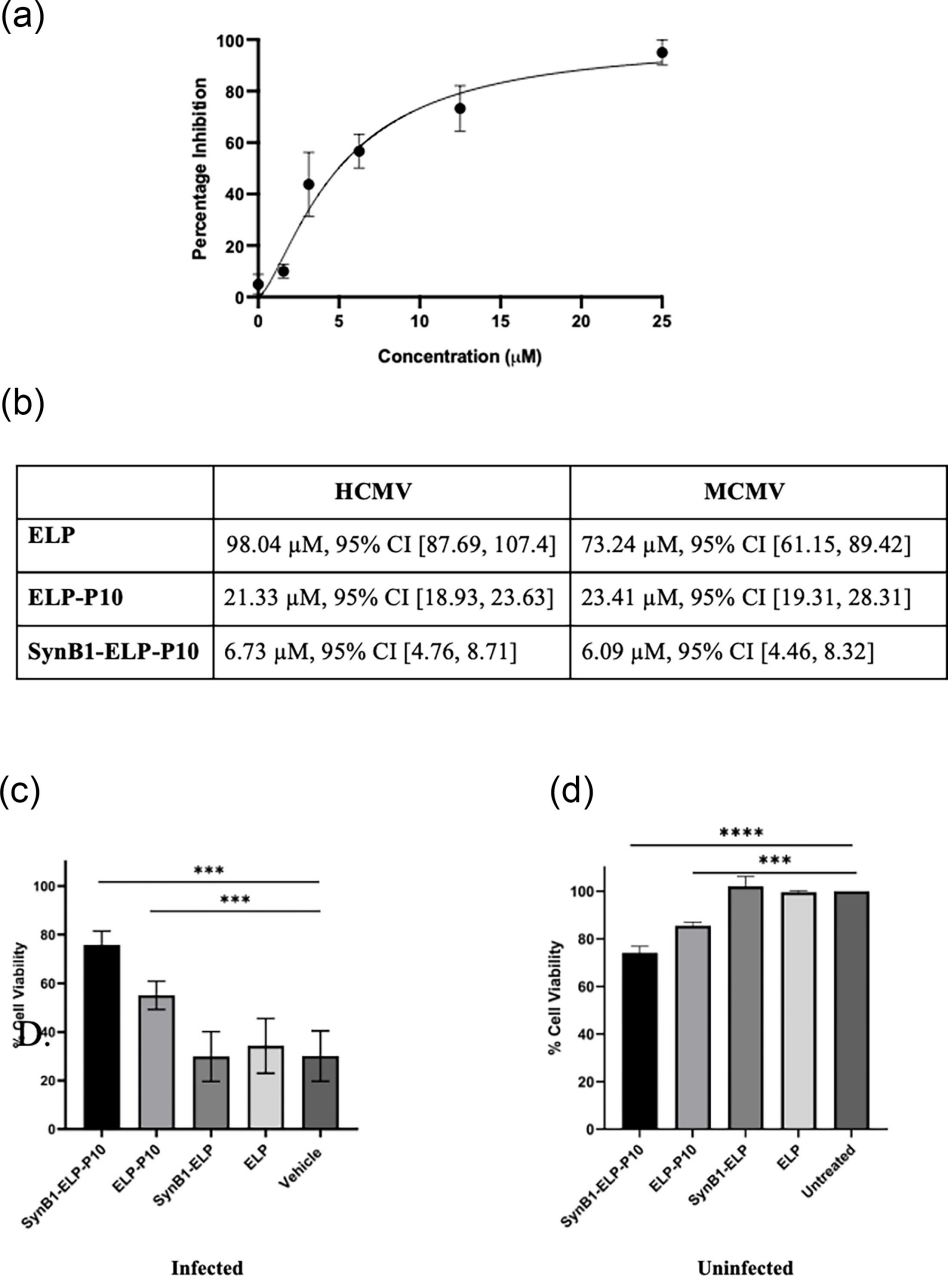
(**a**) Assessing the inhibitory potential of SynB1-ELP-P10 against MCMV in MEFs: the IC_50_ of SynB1-ELP-P10 against MCMV was assessed by infecting cells with K181 at a low m.o.i. of 0.01 for 1 h, followed by treatment with SynB1-ELP-P10 for 5 days. The % inhibition of virus growth is plotted against the concentration from three experiments performed in triplicate. The IC_50_ of SynB1-ELP-P10 was calculated to be 6.094 µM with a 95% CI [4.461, 8.325]. (**b**) Table summarizing the IC_50_ values of ELP, ELP-P10 and SynB1-ELP-P10 against HCMV and MCMV. Cell viability (%) in (c) MEFs infected with MCMV versus (d) uninfected MEFs, along with different treatment groups. MEFs were infected with MCMV with an m.o.i. of 1 for an hour, followed by treatment with ELP-P10 or SynB1-ELP-P10, along with appropriate controls, or vehicle-treated in triplicate for 5 days. At 5 dpi, cell viability was measured using the MTS assay. The percentage of cell survival was plotted for different treatment groups, and the data were analysed by one-way ANOVA using the Tukey post-hoc test. Asterisk represents *P* value < 0.0001.

To test the antiviral efficacy of SynB1-ELP-P10 in a mouse model, we first sought to determine the antiviral efficacy of SynB1-ELP-P10 against MCMV in cell culture. Because of the species specificity of CMV, MCMV experiments were performed using MEFs. First, the antiviral efficacy of SynB1-ELP-P10 was determined against MCMV in cell culture by determining its IC_50_. The IC_50_ of SynB1-ELP-P10 was calculated to be 6.094 µM with a 95% CI [4.461, 8.325], which approximated its IC_50_ against HCMV (Fig. 4a). This could be attributed to the fact that the CR2 of pp150 is conserved against all beta herpesviruses. Table in Fig. 4(b) summarizes the IC_50_ of ELP-P10 and SynB1-ELP-P10 along with ELP control.

Cell viability was assessed using uninfected and MCMV-infected MEFs to determine any cytotoxic effects of the treatments on MEFs. Following MCMV infection, SynB1-ELP-P10 and ELP-P10 treatments were protective against virus-induced cell death at 3 dpi (Fig. 4c), with SynB1-ELP-P10 treatment showing a significantly higher cell viability (75%) compared to ELP-P10 (55%). Notably, SynB1-ELP and ELP control did not protect the cells from virus-induced lytic cell death, thereby showing reduced cell viability. Cell viability was similar across treatment groups in this assay in the uninfected cells. As seen previously using HFFs, SynB1-ELP-P10 and ELP-P10 treatment exhibited a weak cytotoxic effect on the uninfected MEFs, whereas SynB1-ELP and ELP control caused no cell death (Fig. 4d). Altogether, this demonstrates that SynB1-ELP-P10 can be used as a potent antiviral against MCMV in an *in vivo* mouse model.

### SynB1-ELP-P10 and ELP-P10 localize in the cell cytoplasm

**Fig. 5.**
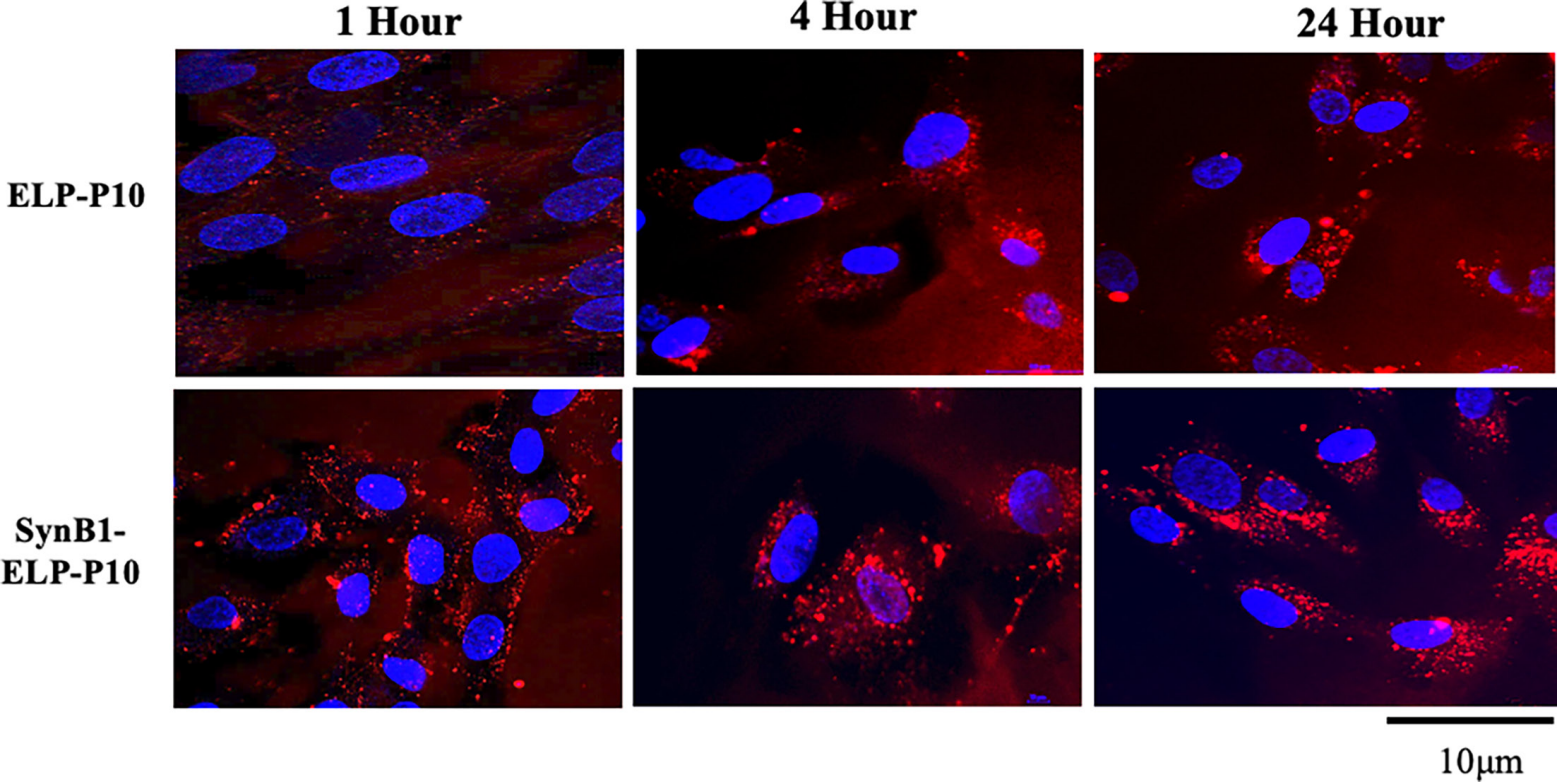
Subcellular localization of rhodamine-labelled SynB1-ELP-P10 and ELP-P10: HFFs were treated with 10 µM of rhodamine-labelled ELP-P10 or SynB1-ELP-P10 (red) for 1 h at 37 °C in triplicates. Cells were fixed 1, 4 and 24 h later with 4% PFA and stained with DAPI (blue). Images were acquired using a laser scanning confocal microscope. Because of the considerable differences in the labelling efficiency of SynB1-ELP-P10 and ELP-P10, the fluorescence intensity of the images does not represent the relative amount of proteins in the cell.

To determine whether the increased inhibitory potential of SynB1-ELP-P10 compared to ELP-P10 is due to enhanced cellular uptake mediated by the CPP or an alternative mechanism, we assessed the localization of rhodamine-labelled SynB1-ELP-P10 and ELP-P10 using confocal fluorescence microscopy at different time points. After a 1 h incubation, intense plasma membrane fluorescence was observed for all polypeptides, with some additional fluorescent polypeptides present in the cytoplasm. After 4 h, the proteins accumulated in the cytoplasm, with a higher SynB1-ELP-P10 accumulation than ELP-P10. Briefly, 24 h later, a punctate cytoplasmic distribution of both polypeptides was observed (Fig. 5). Taken together, these data demonstrate that both polypeptides localize in the cell cytoplasm.

### SynB1-ELP-P10 maintains subcutaneous bioavailability and has increased renal deposition relative to ELP-P10

**Fig. 6.**
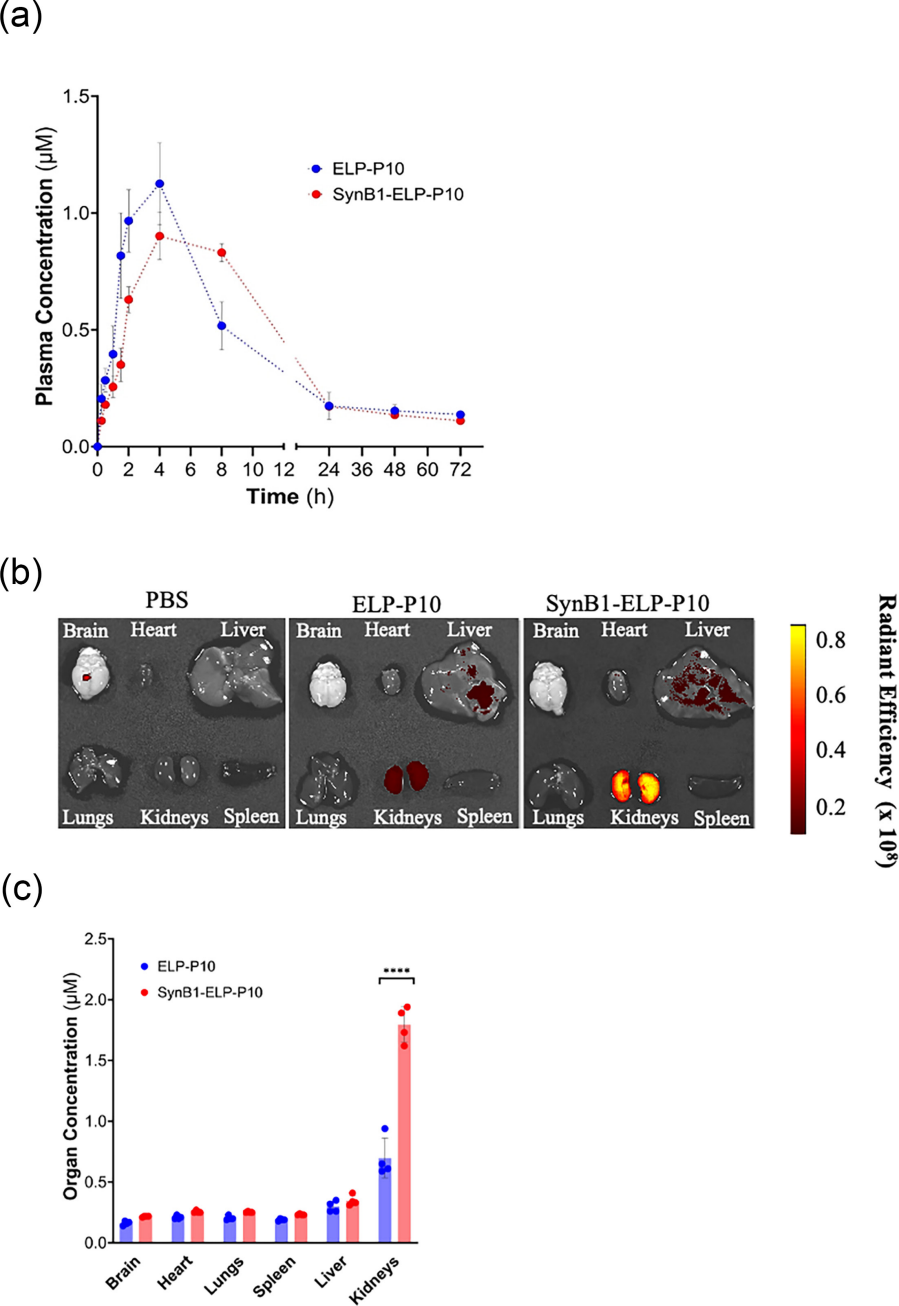
Pharmacokinetics and biodistribution of ELP-P10 and SynB1-ELP-P10: rhodamine-labelled ELP-P10 or SynB1-ELP-P10 was administered by subcutaneous injection. (a) Plasma pharmacokinetics were determined for 72 h after injection. (b) and (c) In a separate cohort of mice, major organ biodistribution was determined 4 h after injection by *ex vivo* whole-organ fluorescence imaging.

To determine if the addition of a CPP improves the pharmacokinetic properties of ELP-P10, the pharmacokinetics and biodistribution of ELP-P10 versus SynB1-ELP-P10 were compared following subcutaneous administration of 500 nmol Kg^−1^ of rhodamine-labelled ELP-P10 and SynB1-ELP-P10. Direct measurement of plasma fluorescence revealed that both proteins were rapidly detected in plasma within the first hour, and both ELP-P10 and SynB1-ELP-P10 displayed similar pharmacokinetic and biodistribution profiles, indicating that fusion with the CPP did not significantly change the pharmacokinetic parameters (Fig. 6a). Biodistribution analysis performed through whole-organ *ex vivo* imaging of harvested tissues 4 h post-injection revealed that both proteins accumulated at low levels in the brain, heart, lungs, spleen and liver without marked differences, but SynB1-ELP-P10 consistently showed a trend towards higher accumulation across these organs (Fig. 6b). However, these differences did not reach statistical significance. SynB1-ELP-P10 exhibited significantly higher accumulation in the kidneys, reaching ~1.8 µM compared to lower levels observed with ELP-P10 (0.7 µM). Together, these results demonstrate that incorporation of the CPP substantially improved the accumulation of ELP-P10 in the kidneys. This feature could be particularly valuable in kidney transplant settings where HCMV poses a significant risk, positioning SynB1-ELP-P10 as a promising candidate for targeted antiviral strategies in transplant medicine.

### SynB1-ELP-P10 treatment led to a reduction in virus titres in MCMV-infected mouse organs

**Fig. 7.**
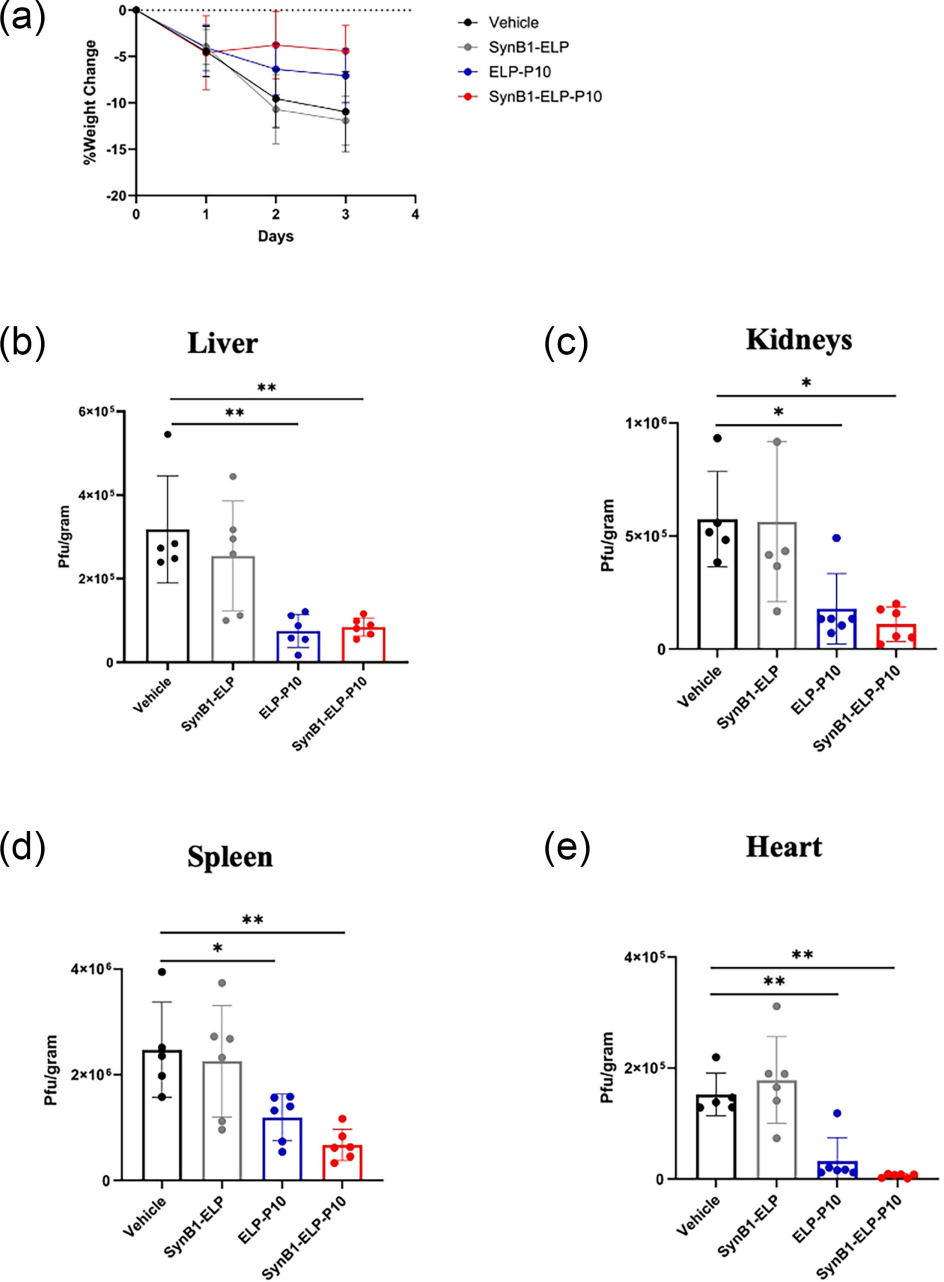
Efficacy of SynB1-ELP-P10 and ELP-P10 in MCMV-infected mice: mice (BALB/c, 3 males and 3 females per treatment group) were infected intraperitoneally with 10^6^ p.f.u. ml^−1^ of MCMV (K181 strain), followed by treatment with 1,000 nmol Kg^−1^ of SynB1-ELP-P10, ELP-P10 or SynB1-ELP-GGC (control) subcutaneously 1 h after infection. Mice were sacrificed on day 4 post-infection, and vital organs were harvested for plaque assay. (**a**) Percentage change in the body weight. Viral load in MCMV-infected mouse organs: (b) liver, (**c**) kidneys, (**d**) spleen and (e) heart, with different treatments assessed by plaque assay. Data were analysed in GraphPad Prism with one-way ANOVA; an asterisk represents a *P* value<0.05.

To further investigate whether the enhanced biodistribution properties of SynB1-ELP-P10 translated into improved antiviral activity, BALB/c mice were intraperitoneally infected with 10^6^ p.f.u. of MCMV K181. At 1 h post-infection, mice were subcutaneously administered 1,000 nmol Kg^−1^ (determined from PK and BD data) of ELP-P10/SynB1-ELP-P10/SynB1-ELP (control) or vehicle-treated. Mice were treated with the same dose of drugs every 24 h (based on the PK data), and their weights were monitored. At 3 dpi, animals were euthanized, and the liver, kidneys, spleen and heart were harvested and used for plaque assay to enumerate the viral titres in the infected organs. Compared to vehicle and SynB1-ELP treatment, the percentage change in body weight following ELP-P10 and SynB1-ELP-P10 was less, with SynB1-ELP-P10 showing a greater protective effect, although not statistically significant (Fig. 7a). As observed previously with MCMV infection following IP infection, a high viral load was observed in all major organs except the lungs. Both ELP-P10 and SynB1-ELP-P10 treatment led to a significant reduction in the viral titres in all vital organs compared to vehicle or control (SynB1-ELP) treatment (Fig. 7). Following ELP-P10 treatment, viral titre was reduced by 76% in the liver (Fig. 7b), 65% in the kidney (Fig. 7c), 52% in the spleen (Fig. 7d) and 79% in the heart (Fig. 7e). While SynB1-ELP-P10-treated mice exhibited a trend towards lower viral titres in key target organs, including the spleen, liver and kidneys, compared to ELP-P10-treated mice, these differences did not reach statistical significance. SynB1-ELP-P10 led to a 73% reduction in the viral load in the liver, 80% in the kidneys, 73% in the spleen and 96% in the heart compared to the vehicle treatment. Clinical observations, including weight change and disease symptoms, similarly showed no significant difference between ELP-P10 and SynB1-ELP-P10 treatment groups, though mice receiving SynB1-ELP-P10 displayed a tendency towards improved outcomes relative to ELP-P10. Collectively, these findings indicate that both proteins are effective against MCMV *in vivo*, and while the CPP-modified ELP-P10 shows a trend towards enhanced potency, further optimization of dosing or timing may be required to achieve statistically significant improvements over ELP-P10.

### Peripheral immune cell analysis revealed no significant differences among treatment groups

**Fig. 8.**
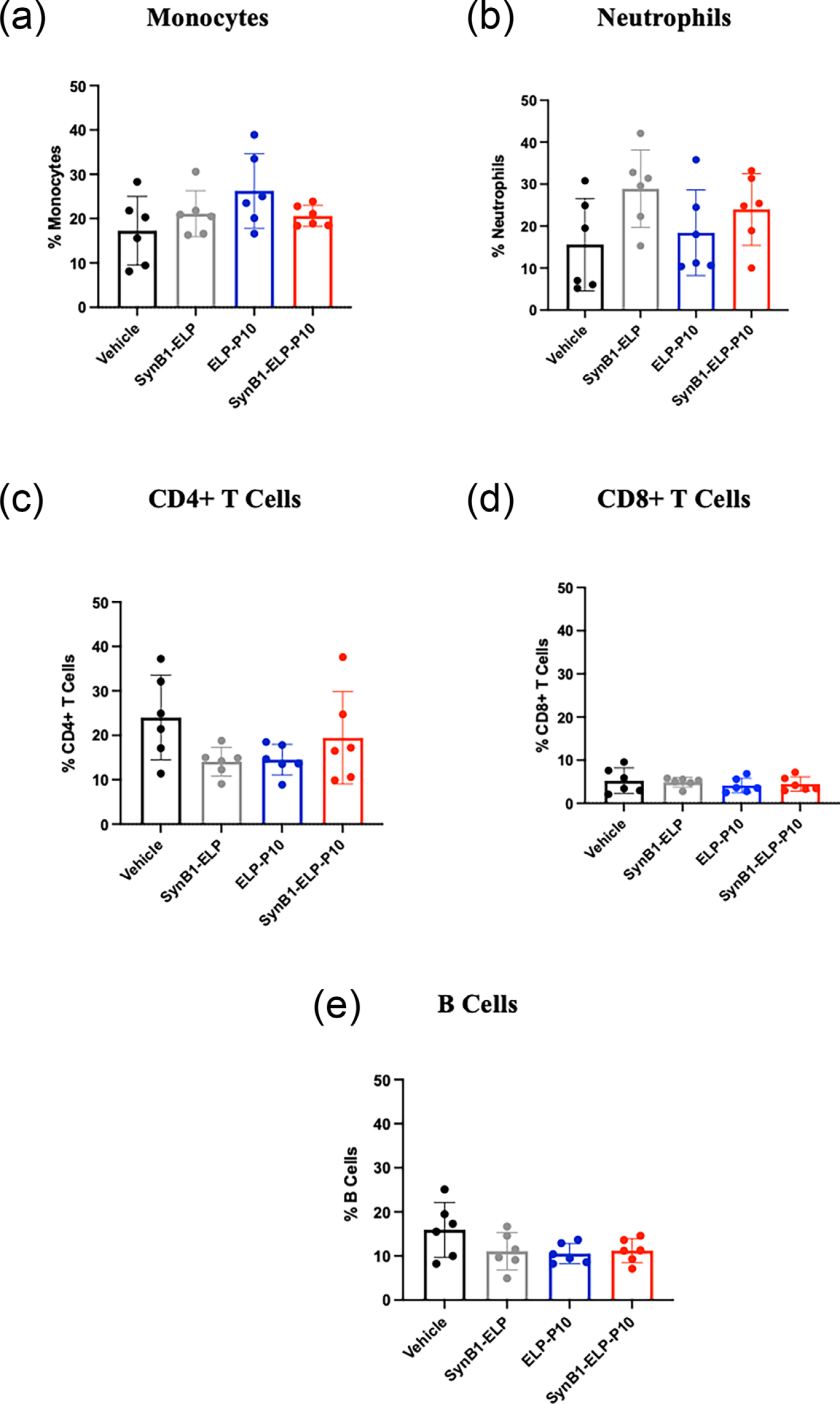
Assessment of peripheral immune cell analysis following ELP-P10 and SynB1-ELP-P10 treatment in MCMV-infected mice: blood collected from the animals at the conclusion of the efficacy study was used for immune cell analysis. The PBLs were stained with a cell viability stain, followed by an FcR block, and then stained with antibodies to identify (a) monocytes, (b) neutrophils, (c) CD4^+^ T cells, (d) CD8^+^ T cells and (e) B cells, and then analysed by flow cytometry. The percentage of different cell types was plotted for different treatment groups, and data were analysed in GraphPad Prism using one-way ANOVA.

To investigate whether ELP-P10 or SynB1-ELP-P10 treatment led to any strong immune activation or suppression at the level of circulating immune cells, peripheral immune cell analysis was performed using the blood collected at day 3 post-infection from MCMV-infected mice, treated with vehicle, SynB1-ELP, ELP-P10 or SynB1-ELP-P10. Flow cytometric quantification of PBLs showed comparable proportions of monocytes, neutrophils, B cells, CD4^+^ T cells and CD8^+^ T cells, demonstrating that none of the treatment conditions resulted in detectable alterations in the distribution of major immune cell populations (Fig. 8a–e). This highlights that no strong immune activation or suppression was observed at the level of circulating immune cells following ELP-P10 and SynB1-ELP-P10 treatment.

## Discussion

Our study demonstrates successful inhibition of HCMV as well as MCMV using the novel biologic SynB1-ELP-P10. Our previous study has shown the effectiveness of the ELP-conjugated anti-CMV inhibitory peptide (ELP-P10) against CMV [48]. To address the limitation of the higher IC_50_ associated with ELP-P10, in this study, the CPP SynB1 was added to the N terminus of ELP-P10. SynB1-ELP-P10 inhibits HCMV and MCMV growth at a lower IC_50_ due to increased cellular uptake, making it a suitable candidate to be used as an effective antiviral against CMV infection.

The conserved nature of CR2 in all the beta herpesviruses supported its utility against HCMV and provided a rationale for extending its use to MCMV models, thereby enabling preclinical evaluation *in vivo* [42]. Therefore, we first determined the antiviral efficacy of ELP-P10 against HCMV. As shown in Fig. 2(a), ELP-P10 inhibited HCMV growth with an IC50 of 21.33 µM. While the unconjugated P10 has an IC50 of 1.33 µM, ELP-P10 has ~20-fold higher IC50, indicating reduced potency [39,48]. This reduction can be attributed to several factors, including differences in the uptake of a small peptide (P10) versus a larger protein (ELP-P10), as higher molecular weight often limits the cellular uptake. Another reason could be the steric hindrance from the large size of the ELP carrier, which could impair the ability of P10 to interact with the capsid binding proteins and inhibit the virus growth. This highlights a trade-off between the potency and pharmacological properties. As shown in our previous study, ELP-P10 has better pharmacokinetics and biodistribution compared to unconjugated P10. Although P10 has higher potency *in vitro*, it exhibits poor pharmacokinetic properties, such as short half-life and rapid clearance in an *in vivo* system [48]. Therefore, the apparent loss in potency can be compensated by the enhanced pharmacokinetic properties offered with ELP conjugation. As seen previously with our MCMV data, ELP control inhibits HCMV at an IC50 of 98.04 µM, which is significantly higher compared to ELP-P10. The ELP control-treated group produced significantly larger plaques compared to the smaller plaques in the ELP-P10-treated group. The smaller-sized plaques indicate a reduction in cell-to-cell virus spread in foci, inferring a reduction in virus growth. This non-specific inhibition could be a result of the large size and doses of ELP on the cellular endocytosis or exocytosis. Future optimizations could include modifying the size of the ELP moiety to balance the potency versus pharmacokinetics [57,58]*.*

To address the limitation associated with the high IC_50_ of ELP-P10, the CPP SynB1 was added to the N terminus of ELP-P10 to enhance its cellular uptake. Therefore, to test our hypothesis that the addition of SynB1 will enhance the potency of ELP-P10, we determined the antiviral activity of SynB1-ELP-P10 against HCMV. As shown in Fig. 3(b), SynB1-ELP-P10 inhibits HCMV growth with an IC_50_ of 6.73 µM, which is threefold more potent than ELP-P10. This could be attributed to the fact that SynB1 is known to enhance the cellular uptake of its cargo molecule into the cell [59]. The addition of SynB1 ensures that a larger proportion of the therapeutic reaches the intracellular environment, thereby increasing its availability to exert its function.

CPPs are known to improve the pharmacokinetics and biodistribution profiles of the attached therapeutic via several mechanisms [60]. CPPs facilitate cellular uptake and intracellular delivery, reducing the rapid clearance from the body by enhancing tissue accumulation and retention [60]. Moreover, the charged nature of some CPPs allows them to interact with the cell/tissue membrane, thereby enhancing the translocation of the therapeutic across the biological barrier [49,51,61]. As expected, SynB1-mediated uptake of ELP-P10 allowed it to accumulate more efficiently in target tissues, as observed with the preferential kidney accumulation of SynB1-ELP-P10, where higher intracellular levels were achieved relative to the unconjugated ELP-P10. While such kidney-targeted distribution raises the possibility of enhanced antiviral efficacy in renal tissue – a clinically relevant site in the context of kidney transplantation – it also warrants further evaluation to exclude potential nephrotoxicity during prolonged administration [62]. But a previous study using prolonged administration of another ELP-delivered therapeutic, ELP-VEGF, in a rat model has shown that ELP-conjugated vascular endothelial growth factor (VEGF) does not induce toxicity in the therapeutic dose range, and doses one hundred times higher than the therapeutic dose were needed to observe any adverse signs in rats [63].

Evaluation of ELP-P10 and SynB1-ELP-P10 in the murine model of CMV infection provided critical insight into the impact of SynB1 addition on therapeutic activity. Both ELP-P10 and SynB1-ELP-P10 demonstrated a significant reduction in the viral load compared to controls, confirming their *in vivo* efficacy. Consistent with our previous study, ELP-P10 treatment led to a significant reduction in viral load in the infected organs. We observed a trend towards more inhibition with SynB1-ELP-P10 compared to ELP-P10; however, these differences did not reach statistical significance. There is a possibility that the intrinsic antiviral potency of ELP-P10 and SynB1-ELP-P10 *in vivo* may be similar. Another possibility is that the viral challenge dose used for *in vivo* efficacy studies may have been too high to discern differences between the two proteins. Mice were infected with a relatively high dose of MCMV (10^6^ p.f.u.), which likely results in robust viral replication. Even though SynB1-ELP-P10 is pharmacologically active and able to exert its antiviral effect, the overwhelming viral burden may have masked more subtle differences in potency between ELP-P10 and SynB1-ELP-P10. In other words, the therapeutic effect may have been partially saturated by the magnitude of viral replication occurring in the organs, such that further improvements in biodistribution or cellular uptake provided by SynB1 conjugation were insufficient to translate into statistically significant reductions in viral titres. Future studies using lower or more physiologically relevant inoculation doses may help to better delineate the comparative efficacy of ELP-P10 and SynB1-ELP-P10 *in vivo*. Interestingly, despite the low accumulation of the therapeutic in the other organs such as liver, spleen and heart, a significant reduction in viral titres was observed. This suggests that the prolonged systemic bioavailability of SynB1-ELP-P10 allowed for exposure of these tissues to our therapeutic, which, even at sub-IC_50_ levels, might contribute to inhibition in virus growth. To achieve higher inhibition with SynB1-ELP-P10, strategies including dose adjustment or prolonged treatment could be employed.

Altogether, these results demonstrate a significant reduction in HCMV as well as MCMV growth using SynB1-ELP-P10. This suggests that SynB1-ELP-P10 can be used as an effective antiviral strategy against CMV infection. An important translational consideration is the potential use of ELP-P10 and SynB1-ELP-P10 in the context of cCMV infection. Many small-molecule antivirals and biologics can cross the placental barrier, raising concerns about foetal drug exposure and potential developmental toxicity. In contrast, ELPs do not cross the placental barrier as they are not the substrate for neonatal Fc receptors [58]. This property may offer a therapeutic advantage in pregnancy-associated CMV infections, where maternal treatment could suppress viral replication without significant risk of direct foetal exposure to the therapeutic. In the context of cCMV infection, early therapeutic intervention in the mother can be a key strategy to limit maternal viraemia and thereby reduce the risk of vertical transmission to the foetus. Because ELP-based peptide therapeutics can be administered safely without the risk of foetal exposure, they are particularly attractive for use early in pregnancy, when preventing viral spread from the mother to the foetus is most critical. Additionally, future studies in the lab are focused on developing ELPs that can be transported across the placenta, making the use of ELP-conjugated therapeutics feasible to treat congenital infections.

## Abbreviations

ACV: acyclovir
cCMV: congenital cytomegalovirus
CDV: cidofovir
CI: confidence interval
CMV: cytomegalovirus
CPPs: cell-penetrating peptides
CR: conserved region
DMEM: Dulbecco’s modification of Eagle’s medium
dpi: days post-infection
ELP: elastin-like polypeptide
FOS: foscarnet
GCV: ganciclovir
HCMV: human cytomegalovirus
HFFs: human foreskin fibroblasts
HIV: human immunodeficiency virus
IC_50_: half-maximal inhibitory concentration
MCMV: murine cytomegalovirus
MEFs: mouse embryonic fibroblasts
PBLs: peripheral blood leucocytes
VEGF: vascular endothelial growth factor
